# Prescribing practices of primary-care veterinary practitioners in dogs diagnosed with bacterial pyoderma

**DOI:** 10.1186/s12917-014-0240-5

**Published:** 2014-10-08

**Authors:** Jennifer F Summers, Anke Hendricks, David C Brodbelt

**Affiliations:** Department of Population and Public Health (PPH), Royal Veterinary College, Hatfield, UK; Queen Mother Hospital for Animals (QMHA), Royal Veterinary College, Hatfield, UK

**Keywords:** Pyoderma, Canine, Antimicrobial, EPR, Prescribing, VetCompass

## Abstract

**Background:**

Concern has been raised regarding the potential contributions of veterinary antimicrobial use to increasing levels of resistance in bacteria critically important to human health. Canine pyoderma is a frequent, often recurrent diagnosis in pet dogs, usually attributable to secondary bacterial infection of the skin. Lesions can range in severity based on the location, total area and depth of tissue affected and antimicrobial therapy is recommended for resolution. This study aimed to describe patient signalment, disease characteristics and treatment prescribed in a large number of UK, primary-care canine pyoderma cases and to estimate pyoderma prevalence in the UK vet-visiting canine population.

**Results:**

Of 54,600 dogs presented to 73 participating practices in 2010, 683 (1.3%) had a pyoderma diagnosis recorded in available electronic patient record (EPR) data. Antimicrobials were dispensed in 97% of cases and most dogs were prescribed systemic therapy (92%). Agents most frequently prescribed were amoxicillin-clavulanate, cefalexin, clindamycin and cefovecin. Systemic antimicrobials were prescribed for fewer than 14 days in around 40% of study cases reviewed in detail. Prescribed daily doses were below minimum recommended daily dose (MRDD) in 26% of 43 dogs with sufficient information for calculation of minimum dose.

**Conclusions:**

Antimicrobial prescribing behaviour for treatment of canine pyoderma was variable but frequently appeared inconsistent with current recommendations. Use of clinical data from primary practice EPRs can provide valuable insight into common clinical conditions and associated prescribing.

**Electronic supplementary material:**

The online version of this article (doi:10.1186/s12917-014-0240-5) contains supplementary material, which is available to authorized users.

## Background

Canine pyoderma results from bacterial infection of the skin and associated structures. Presentations can include a wide range of clinical lesions from erythema, alopecia and pruritus to macules, papules, pustules, crusts, collarettes, scaling, deep folliculitis, furunculosis, dermal fistulae, cellulitis, panniculitis and vasculitis. Clinical sub-division of the condition into surface, superficial and deep pyoderma is made according to the depth and extent of bacterial infection present within cutaneous tissue. In the dog, the most commonly isolated causal pathogen is the coagulase positive staphylococcal species known as *S. pseudintermedius*. Other staphylococcal (e.g. *S. aureus, S. schleiferi*) and non-staphylococcal bacteria (e.g. *Escherichia coli*, *Pseudomonas* or *Proteus* species) can also be isolated from affected skin in some cases. While these species can act as primary pyoderma pathogens, particularly in immune-compromised patients, they are usually secondary infectious agents or simply lesion contaminants [1-3]. Canine pyoderma is usually secondary to one or more underlying disease processes which compromise the defence mechanisms protecting the skin from infection [4-7]. Recurrence of pyoderma lesions after successful antimicrobial treatment is common if primary conditions remain undiagnosed or are not appropriately managed.

Clinical consequences for individual affected dogs and the scale of disease-burden at a population level are both of concern when considering the overall impact of pyoderma on canine welfare. The condition causes varying degrees of pain and pruritus depending on depth and extent of associated lesions [8,9]. Skin disease is one of the most common reasons for presentation of pet dogs for veterinary care [10,11], and several reports support the clinical impression that canine pyoderma appears to be a relatively common diagnosis [12-15]. In addition, recent evidence-led guidelines agree that antimicrobial treatment, either topical, systemic or both, is indicated in all but the mildest cases of pyoderma in the dog (i.e. those involving only a small number of extremely localised superficial lesions) [16-18]. Thus, clarification of the impact of canine pyoderma on dog welfare, as well as the level and appropriateness of antimicrobial usage for the condition in the UK is required. Large scale, UK-specific surveys using data from the primary clinical practice setting could provide an estimate of the proportion vet-visiting dogs affected by pyoderma over a specified period of time, and provide insight into the extent and nature of associated antimicrobial prescribing by vets managing these dogs. This information could facilitate evidence-based assessment of the potential impact of the condition in the UK with respect to animal welfare as well as exploring whether veterinary antimicrobial usage is in accordance with current guidelines [18].

This study aimed to use pre-existing electronic patient health record (EPR) data from a large number of UK primary practices to describe the frequency of the condition and the treatment approaches adopted for canine pyoderma as managed by first opinion veterinary practitioners in the UK.

## Results

### Prevalence of diagnosis with pyoderma

A total of 683 dogs with at least one reported diagnosis of pyoderma in the year 2010 were identified using available EPRs from 73 individual practices. The total number of individual dogs with one or more clinical encounters (for any reason) recorded by any of these VetCompass-participating practices during the year 2010 was 54,600. Thus, 1.3% (95% confidence interval (CI) 1.2-1.4%) of all dogs presented for any reason to participating practices in 2010 were diagnosed with pyoderma at least once during that year. Based on in-depth case review in the randomly selected subgroup (see later), a 10% misclassification rate was estimated, suggesting the true prevalence within the study period was approximately 1.1% (95% CI 1.0-1.2%).

### Characteristics of dogs diagnosed with pyoderma

Of all 683 identified cases, 392 (57%) were male. Neutering status at the time of the earliest pyoderma episode in 2010 was clearly recorded in 455 (66.6%) identified cases and 425 (93.4%) of these were neutered. Neutering status at this time was neither recorded nor deducible from available information in the remaining 228 cases. Median age at diagnosis was 5.0 years and ranged from 0.1 – 16.2 years (i.e. between approximately 5 weeks and 16 years 10 weeks). Weight was recorded on the date of diagnosis in 369 study dogs (54.0%) and ranged from 1.2 – 81.5 (median 19.2) kgs. At least one weight value was recorded within 28 days of the date of diagnosis in 478 dogs (70.0%), within six calendar months in 594 dogs (87.0%) and within one year in 629 dogs (92.1%). Of 90 different breeds represented, those accounting for at least 3% of all study dogs were the crossbreed (105 dogs, 15%), Labrador retriever (61, 9%), West Highland White terrier (54, 8%), Staffordshire bull terrier (50, 7%), German shepherd dog (38, 6%), Cocker spaniel (31, 5%), Golden retriever (26, 4%), Yorkshire terrier (24, 4%) and Jack Russell terrier (22, 3%).

### Prescribing of systemic and topical antimicrobial treatment

**Figure 1:**
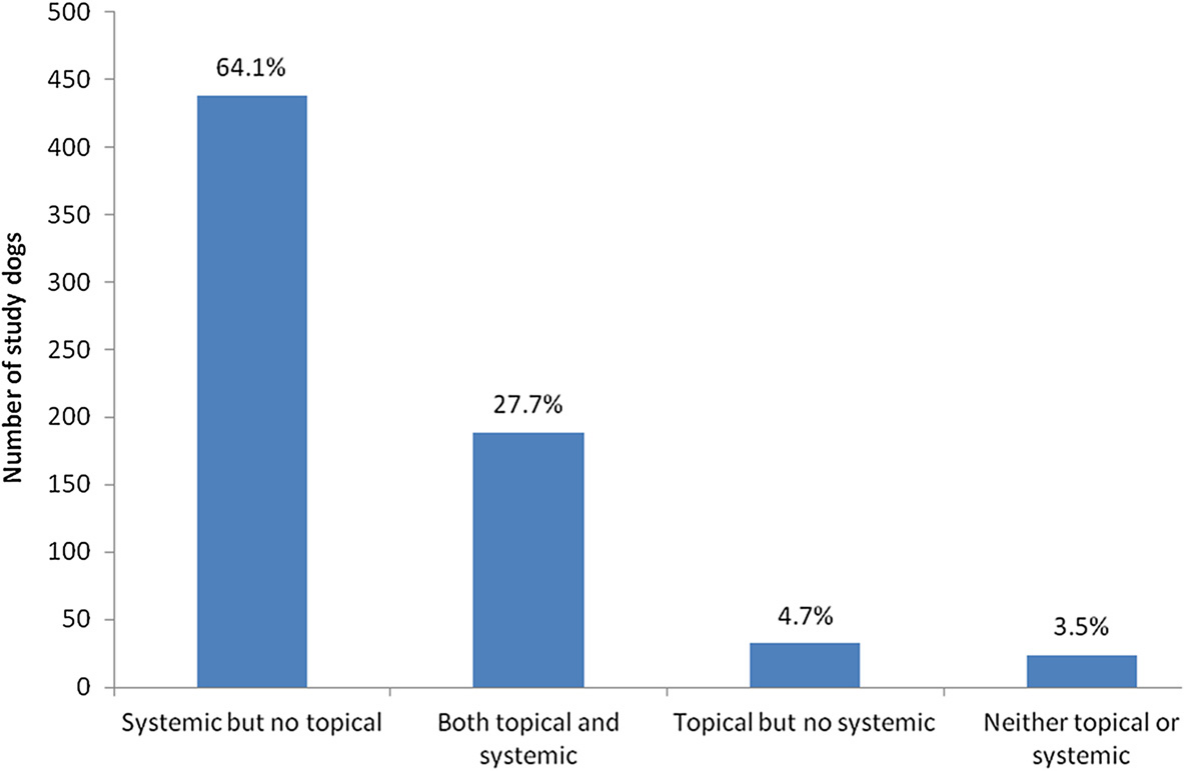
**Antimicrobial formulation type/s initially prescribed by veterinarians treating UK canine pyoderma cases.** Frequency and proportions of all 683 identified canine pyoderma cases prescribed either systemic, topical or both types of antimicrobial agent within 24 hours of their earliest recorded pyoderma diagnosis in 2010.

**Figure 2:**
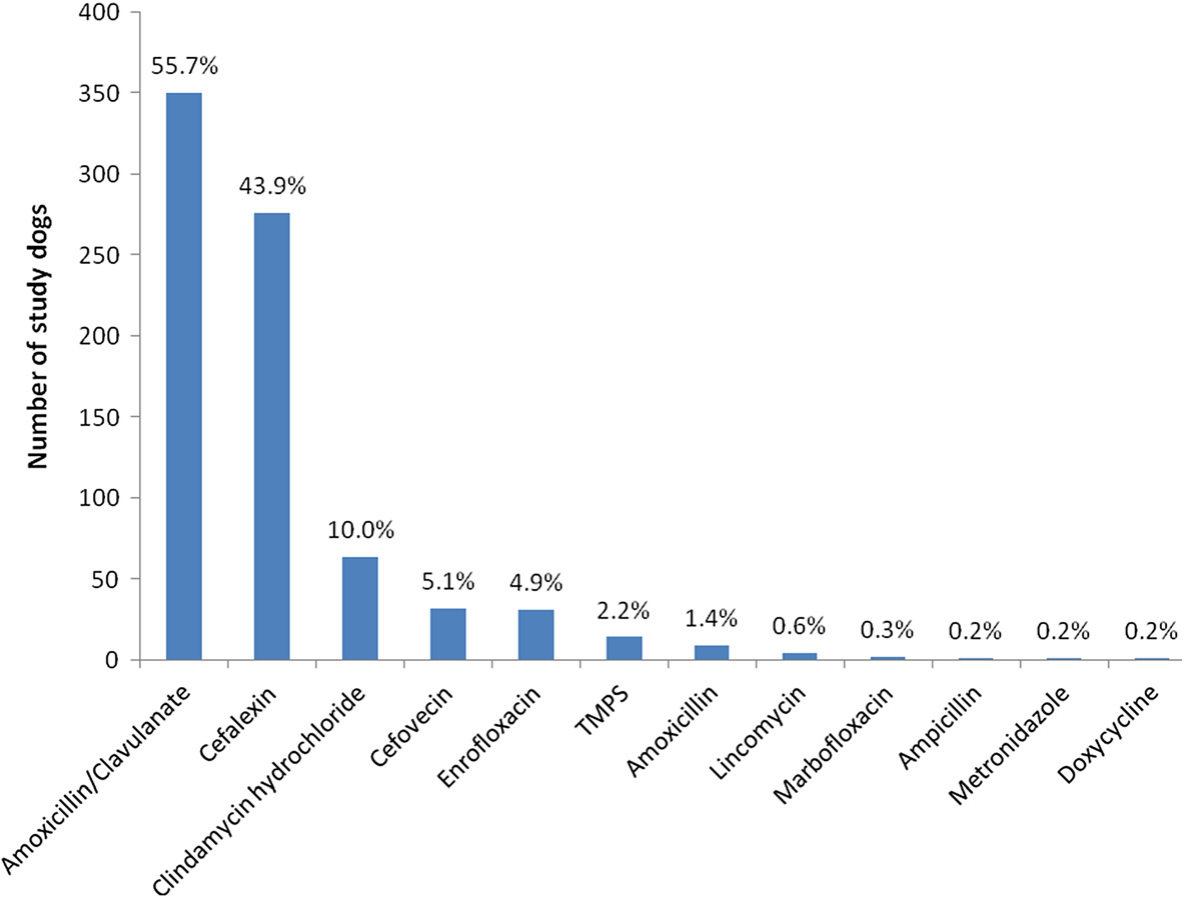
**Systemic antimicrobial agents initially prescribed or administered by veterinary surgeons treating UK canine pyoderma cases.** Prescribing frequency of 12 individual, systemic antimicrobial agents among 628 canine pyoderma cases within 24 hours of earliest recorded pyoderma diagnosis in 2010 (showing the percentage of these 628 dogs prescribed each agent; percentages do not add to 100% as some dogs were prescribed more than one agent at this time; TMPS, trimethoprim sulphonamide).

**Figure 3:**
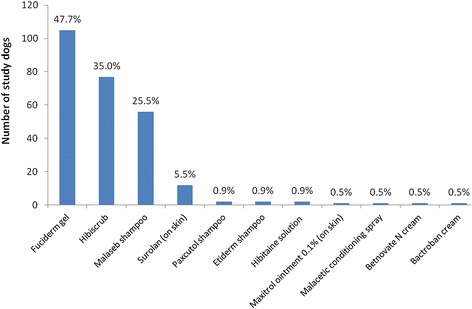
**Prescribing frequencies of individual topical antimicrobial products among study dogs.** Prescribing frequency of individual topical antimicrobial products among the 220 study dogs prescribed at least one topical antimicrobial within 24 hours of earliest pyoderma diagnosis in 2010. The percentage of all 683 study dogs prescribed each combination are shown above the relevant bar. Commercial details (manufacturer and location) for products shown are provided in Additional file 1.

Within 24 hours of the diagnosis of pyoderma, 659 cases (96.5%) were prescribed at least one antimicrobial product for administration systemically and/or topically; most dogs (91.9%) were prescribed at least one systemic antimicrobial, either alone (64.1%) or in combination with a topical product (27.7%). Few dogs were prescribed a topical product only, or no antimicrobial treatment at all (Figure 1). Agents most frequently prescribed for systemic antimicrobial treatment were amoxicillin-clavulanic acid (350 dogs, 55.7% of the 628 dogs prescribed a systemic antimicrobial), cefalexin (276 dogs, 43.9% of 628) and clindamycin (63 dogs, 10.0% of 628) (Figure 2). In total 220 study dogs (32.2%) were prescribed at least one topical antimicrobial product. Products most frequently prescribed were Fuciderm gel (active ingredient fusidic acid; Dechra Veterinary Products Limited; 105 dogs, 47.7% of 220 dogs), Hibiscrub (chlorhexidine; Molnlycke Health Care; 77 dogs, 35.0% of 220 dogs) and Malaseb shampoo (miconazole and chlorhexidine; Dechra Veterinary Products Limited; 56 dogs, 25.5% of 220 dogs) (Figure 3).

Of the subset of 100 study dogs randomly selected for detailed case review ten were excluded from further description due to confirmed (or very likely) violation of study inclusion criteria: chronological review of all available free-text clinical history undermined the certainty of pyoderma diagnosis in eight dogs, and indicated that the episode identified was not newly diagnosed in 2010 in two more. Thus all available EPR information (including free-text) was reviewed in further detail for 90 study dogs, from 29 individual clinics. Pyoderma lesion depth was classifiable in 50 of the retained cases, with superficial lesions the most frequent lesion depth category, reported in 37/50 (74.0%) of these dogs (Table 1). Eighty two (91%) of the 90 cases reviewed in detail had been prescribed at least one systemic antimicrobial in association with the pyoderma episode of interest. In all cases antimicrobials were dispensed at the practice, with no evidence of clients being provided with a written prescription to obtain antimicrobials from elsewhere. In 2 of 82 cases (2.4%) there was evidence that bacterial culture and antimicrobial sensitivity testing were performed during the pyoderma episode studied; in both cases results were available before the prescribed course of antimicrobial treatment commenced. Test results (indicating no bacterial growth from pyoderma lesion swabs) were available in the records of one dog (prescribed cefalexin), and were absent from the records of the other (prescribed cefovecin).

**Table 1:** **Summary of available data on depth of pyoderma lesions in the 90 episodes reviewed in detail**

**Stated depth of pyoderma lesions**	**Number of dogs**	**% of 90 dogs reviewed in detail**
Surface	2	2.2
Superficial	37	41.1
Deep	6	6.7
Mixed (deep + other)	5	5.6
*Unclear/ambiguous/absent clinical description*	*40*	*44.4*
***Total***	***90***	***100***

**Figure 4:**
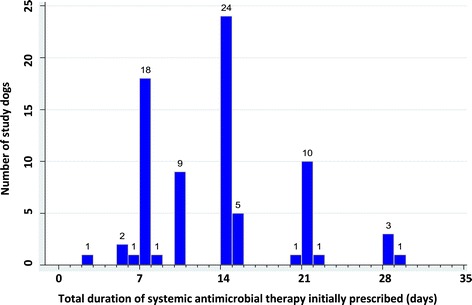
**Total estimated duration of treatment with the initially prescribed systemic antimicrobial agent in study cases.** Total duration of treatment with the initially prescribed systemic antimicrobial agent (assuming medication dispensed was administered as directed) in 77 of the 82 study cases reviewed in detail, prescribed at least one systemic antimicrobial in association with their earliest pyoderma diagnosis in 2010 and with sufficient data available to calculate theoretical duration of prescribed treatment.

Complete information on prescribed daily dose for administration was recorded in 80 (97.6%) of the 82 cases prescribed at least one systemic antimicrobial agent. Median theoretical total duration of treatment with the initially prescribed systemic antimicrobial agent was 14 days (range 1–30 days) and 40.3% (31/77) of the dogs with available course duration information were prescribed treatment for a period of less than 14 days (Figure 4). Prescribed daily doses were considered equivalent to the minimum MRDD in 32 dogs (74% of the 43 dogs with sufficient information available for daily dose calculations), while the remaining 11 (26%) were prescribed doses below the recommended level. In 39 cases it was not possible to determine the MRDD; this was primarily due to absent weight data, but in 3 dogs prescription of multiple systemic antimicrobial agents concurrently prevented assessment of a single primary agent.

Eight (9%) of the 90 dogs in the subset with pyoderma were prescribed topical antimicrobial agents only. Available instructions for administration of topical antimicrobial agents in these dogs did not indicate (or allow calculation of) the intended duration of prescribed antimicrobial therapy, or a theoretical date for treatment completion. Shampoos or liquid washes were prescribed concurrently with antimicrobial treatment in 34/90 (37.8%) dogs; Malaseb (active ingredients miconazole and chlorhexidine) was prescribed for 18 dogs and either Hibiscrub, Hibitaine (active ingredient chlorhexidine) or generic chlorhexidine were prescribed for 16 others.

## Discussion

This study explores the prevalence and treatment of canine pyoderma in a population of UK dogs presented to primary care veterinary clinics during a single year, based on EPR data recorded by vets during case management. Existing studies describing the frequency of bacterial skin infections in dogs presented for veterinary care are sparse, have used a variety of case definitions for pyoderma, and were based on canine populations from different countries [10-13]. Similarly, while studies reporting prescribing patterns of clinicians treating canine pyoderma in primary practice are available, they are often relatively small in scale, not based on UK data, or describe self-reported theoretical prescribing behaviour rather than objectively reporting therapeutic agents dispensed in canine pyoderma cases diagnosed in practice [19-22].

Using available electronic data from participating primary practices it was estimated that approximately 1.1-1.3% of all dogs presented during 2010 were diagnosed with pyoderma at least once during that year. However, the strict case definition applied in this study, as well as the transient, often recurrent nature of the condition suggest that the level of pyoderma in this population annually or at any given point in time is likely to be higher than these estimates. It is particularly relevant to note that the requirement for use of the specific term ‘pyoderma’ as part of the criteria for inclusion of a dog as a study case may have been a particularly important factor in underestimation of true pyoderma frequencies. Based on the data reviewed, a wide range of terms appear to be used by UK veterinary practitioners to describe canine dermatological infections, and diagnostic terms such as “bacterial dermatitis”, “bacterial folliculitis” or variants of “staphylococcal skin infection” are likely to indicate conditions consistent with clinical pyoderma in many cases. However, it was not possible to confirm this assumption with sufficient consistency and confidence using the available data, hence the decision to implement a strict case definition and include only those dogs where recorded diagnosis included the specific term “pyoderma”. While it may have been interesting to search EPR data for each of the potential individual synonyms or equivalent terms for pyoderma (in parallel with identifying cases based on use of the specified clinical term) this was not attempted in the current study due to time constraints. An additional cause of potential prevalence underestimation relates to the frequent lack of recording of a diagnosis within clinical fileds: a diagnosis of any kind was not always stated in dogs presented with skin lesions suspicious of pyoderma, even if antimicrobials were dispensed in association with clinical examination of the skin. Existing prevalence estimates based on dogs presented for primary practice care in the UK and USA are approximately three times higher [10,11], however validity of comparisons with the estimate from this study is limited as these surveys were based on different methodologies for calculation of disease frequency measures. It is possible that the true prevalence of pyoderma in the UK pet dog population does lie somewhere between the estimate in the current study and those previously reported. However, it is important to note that 8 of the 10 ‘cases’ excluded from the current study after detailed clinical data review were discarded due to violation of inclusion criteria. This suggests that the initial search strategy used to identify pyoderma cases in this study was not sufficiently accurate to reliably identify cases; this and other potential study limitations are discussed in more detail later in this report. Antimicrobial treatment was dispensed or administered at the time of diagnosis in most pyoderma cases identified. Studies reporting antimicrobial prescribing patterns specific to canine pyoderma are not available for direct comparison, however this finding is consistent with results of existing surveys from the UK and New Zealand reporting antimicrobial prescribing in companion animal dermatology cases [10,20] and surveys of theoretical prescribing behaviour for canine pyoderma from the UK and Australia [22,23]. These findings are in accordance with current recommendations for successful treatment of the condition, which agree that antimicrobial treatment is generally indicated to achieve clinical resolution of lesions [8,17,18].

Recent, evidence-based guidelines for prudent, effective management of canine pyoderma emphasize the importance of selecting appropriate antimicrobial therapy (i.e. an effective agent administered at an adequate dose rate, dosing frequency and course duration) alongside accurate diagnosis of lesion depth and management of any underlying diseases [8,16]. Choice of antimicrobial delivery in the current study appears to be in line with advice advocating systemic antimicrobial treatment for most superficial and all deep pyoderma lesions in dogs, with concurrent topical antimicrobials or anti-septics useful as adjunctive therapy [9,16,17,24-26]. While systemic antimicrobial therapy alone was most common, around a third of cases were prescribed topical and systemic antimicrobials concurrently. A survey of theoretical prescribing behaviour among Australian clinicians also found that most would prescribe systemic antimicrobials and an antibacterial shampoo for therapy of deep pyoderma in dogs [23].

The most frequently prescribed systemic antimicrobial agents in the present study were amoxicillin-clavulanic acid, cefalexin (broad-spectrum beta lactams) and clindamycin, all of which are considered appropriate first-line choices for treatment of straightforward superficial and surface pyoderma in dogs [16,27]. This finding is consistent with reports from various countries, including the UK, describing antimicrobial prescribing in companion animals generally, for dermatological complaints and specifically for treatment of canine pyoderma [10,19-21,28-33].

Bacterial culture and antimicrobial sensitivity testing was recorded as part of management in only 2% of all study cases reviewed in detail. Low levels of C&S testing have also been reported in other studies describing general antimicrobial prescribing in companion animals, and specifically for treatment of canine skin infections [19,20,29]. In the present study enrofloxacin and cefovecin were each prescribed in approximately 5% of cases. Empirical and first line use of fluoroquinolones or the 3rd generation cephalosporin cefovecin remains controversial for treatment of canine pyoderma, though it can be useful when difficulties with oral administration of treatment are experienced or foreseen [16,33,34]. Thus, the findings of the present study could reflect frequent empirical selection of these agents for canine pyoderma in primary practice. It is, however, important to remember that antimicrobial choice may be dictated by factors relating to practicality of administration, compliance or previous adverse drug reactions and assessment of information justifying reasons for drug choice was beyond the scope of this study.

Delivery of a sufficiently high dose of the chosen agent, at appropriate intervals, is recommended to avoid rapid reappearance of lesions while minimizing exposure of bacterial populations to ineffective levels of antimicrobial drugs [16,17]. Traditionally veterinary surgeons have been advised to prescribe antimicrobials for 7–14 days beyond clinical resolution, as confirmed by physical or cytological examination, though this recommendation does not appear to be evidence-based. Consistently sub-therapeutic dosing can lead to poor clinical response, necessitating further antimicrobial courses and encouraging selection of antimicrobial resistant bacterial populations [35]. In this study, around 25% of systemically-treated study dogs reviewed in detail were prescribed doses below those recommended to achieve therapeutic levels (minimum MRDD). In addition, the median treatment duration often appears relatively short, with antimicrobials prescribed for less than 14 days in around 40% of cases, even taking into account the likely predominance of superficial over deep pyoderma cases in the study population. Other studies report similarly short, mean treatment periods for canine pyoderma cases in Finland [21] and New Zealand [20]. Limitations related to the primary practice setting could curtail treatment duration regardless of the preferred clinical approach of the managing practitioner. Economic restrictions, client preferences and failure to attend for re-examinations could result in shorter treatment courses than would be prescribed based on clinical judgement alone. In addition, the study design did not allow evaluation of client compliance with oral dosing instructions; inconsistent or under-dosing is reportedly not uncommon in companion animal practice [36,37]. Therefore, it is possible that the prescribing-focussed findings reported here overestimate the amount and duration of oral antimicrobial treatment actually received by study dogs prescribed systemic antimicrobials at or above the MRDD [16].

The study approach was subject to certain inherent limitations related to the nature of the data used and canine pyoderma as a clinical condition. The retrospective data analyzed were not collected primarily for research purposes, thus researchers had no influence on the content, level of pyoderma-related detail or note-taking style of clinical entries and were reliant on the clinical opinion of the examining vet with respect to pyoderma diagnosis and progress assessment. The study case definition for pyoderma required diagnosis to be based on clinical examination by a veterinarian and recorded using specified clinical terminology. However, standardised coding of diagnoses was not consistently applied and the frequency of typing errors, abbreviation and synonym use as well as misspelling of clinical terms made case-searching using the keyword ‘pyoderma’ alone ineffective. The broader search strategy employed improved the sensitivity of the search strategy by capturing as many pyoderma cases as possible, while maintaining strict inclusion criteria to achieve a reasonable level of diagnostic certainty and specificity. This method was more time-consuming and still provided scope for misclassification, i.e. ‘false negatives’ (no explicit statement of pyoderma diagnosis, or diagnosis expressed in terms inconsistent with the case definition) and ‘false positives’. In addition, it appeared that few cases underwent confirmatory diagnostic testing beyond physical examination. Nonetheless, detailed review of a sample of cases did enable quantification of the likely level of uncertainty and misclassification within the dataset and the majority of identified cases probably involved genuine pyoderma. While it is difficult to quantify the true extent of pyoderma misdiagnosis in this study, the condition does generally lend itself readily to diagnosis by simple clinical examination [38], suggesting that the reported prevalence of pyoderma in UK dogs on an annual basis is likely to be of an order similar to (or greater than) that reported in this study.

Further limitations included missing or erroneously recorded data of interest; certain data fields were frequently unpopulated, or contained information which proved incorrect based upon full review of clinical notes. Data on specific factors of clinical interest were often absent despite review of all available free-text data entries, limiting comprehensive description of study animals and evaluation of certain variables of interest in the study. A particular example was the frequent absence of recorded data on follow-up for study cases: re-examinations to confirm clinical resolution appeared to occur relatively infrequently, even when advised by the prescribing clinician. Thus it was not possible to describe (or explore factors potentially affecting) clinical response to antimicrobial treatment or recurrence of pyoderma lesions after completion of prescribed therapy. In this study it is likely that the constraints of the clinical setting had a major influence on the data available for clinicians to report as well as the depth of information recorded: in private, primary-care practice non-clinical factors (such as client financial constraints or convenience) often drive decisions on diagnostic approach, case management, as well as the occurrence and timing of any follow-up clinical evaluations. In addition, the time available to busy veterinary surgeons for clinical recording can be limited. However, the data accurately reflect prescribing behaviour of primary practice veterinarians with respect to a larger number of UK canine pyoderma cases than previously described, underlining the potential of EPR data for use in large scale epidemiological studies and practice-based surveillance.

## Conclusions

Approximately 1.1-1.3% of nearly 55,000 dogs attending UK primary practices during 2010 were diagnosed with pyoderma during that year, however this is likely to be a conservative estimate of the true prevalence of the condition in this population at any given time. Study findings suggested that there is scope for improvement with respect to prudent antimicrobial use in canine pyoderma treatment in UK primary-care practice, in terms of empirical selection of certain critically important agents, prescribing of adequate dose rates and particularly sufficient treatment duration. This report highlights the value of practice-based electronic health records for prevalence estimation and description of clinical management of conditions commonly seen in vet-visiting canine populations presented for primary health care.

## Methods

Clinical and animal data from consultations recorded during a single year (2010) were exported for analysis from a database containing all de-identified patient records from dogs attending UK primary-care veterinary clinics participating in the VetCompass Animal Surveillance project [39]. These practices form a convenience sample based on established use of an appropriately configured practice management system (PMS) for clinical record keeping and willingness to contribute clinical data for research purposes. Data from individual practices were extracted centrally via a bespoke clinical reporting query established with the participating practices and groups. Data were uploaded to a File Transfer Protocol (FTP) site, then imported into and stored in the VetCompass structured query language (SQL) database held securely on an RVC server.

Ethical approval was obtained from the Royal Veterinary College (RVC) Ethics and Welfare Committee for the extraction, transfer, secure storage and use of specified data from first opinion patient health records for the purposes of this project^a^. Procedures for data extraction, storage and reporting of analyses were compliant with data protection legislation (P Dron, RVC Data protection officer; Personal communication). Approval for project aims and data collection approach was obtained from the Royal College of Veterinary Surgeons (RCVS). Consent for the use of clinical data was obtained from senior practice managers on behalf of all participating clinics, on the basis that that no animal or client would be identifiable in published findings.

Extracted data fields included patient characteristics (breed, gender, neutering status, date of birth), consultation-related data (date of examination or clinical data entry, free-text clinical notes, any diagnostic codes assigned (VeNom codes [40]) and treatment details (product name and number of items dispensed, dosing instructions from labels generated for dispensed medication). Fields directly identifying participating animals, owners or financial information were not extracted.

Data cleaning and processing was carried out in SQL using a Microsoft Access interface. Participating centres (identifiable in the extracted data to the level of clinic ID number) were assigned unique numerical codes allowing differentiation between clinics in the analysis without compromising participant anonymity. Recorded breed data were standardised according to a pre-existing scheme of canine breed terminology (VeNom codes [40]).

The study case definition for pyoderma required diagnosis to be based on clinical examination by a veterinarian and recorded using specified clinical terminology. Potential study cases were initially identified using the Microsoft Access interface to search for clinical entries where free-text clinical data or diagnostic code fields^b^ contained the word-root ‘pyo’ , with date restrictions applied limiting search results to entries recorded in 2010. All clinical information recorded in the entries highlighted was then reviewed to confirm that the identified ‘pyo’ word-root did appear to indicate an appropriately recorded^c^, positive diagnosis of pyoderma made by a clinician and based on physical examination. It was also confirmed that the diagnosis documented a pyoderma episode newly diagnosed in the year 2010 (i.e. not continuation of an ongoing episode diagnosed the previous year). Dogs with at least one pyoderma episode fulfilling all these criteria were selected for inclusion. In dogs with more than one recorded pyoderma diagnosis during 2010 the earliest recorded diagnosis was selected for description and defined as the pyoderma episode of interest. The start of this episode was defined as the date that diagnosis was first recorded in the PMS clinical records.

Microsoft Access queries were designed to extract fields containing clinical data recorded on the date of diagnosis of the pyoderma episode of interest, product details and amounts (volume or unit number) of medications dispensed or administered on this date, and general signalment data (breed, sex, neutering status) for all selected dogs.

In all study dogs, confirmation of diagnosis with a pyoderma episode in 2010 was initially based only on the earliest free-text clinical entry identified by the search strategy. Similarly description of basic signalment information across all study dogs related only to information extracted from the relevant specific data fields and was not based on review of all available clinical text. Age at diagnosis of the episode of interest was calculated using recorded date of birth and diagnosis date. Where possible, weight data recorded on (or as close as possible to) the date of diagnosis were extracted. Treatment information was extracted from records of item sales occurring within 24 hours of recorded diagnosis of the pyoderma episode of interest. Treatment-related factors included the name (generic and product) and route of administration of the first systemic antimicrobial agent prescribed to treat the identified pyoderma episode. Prescribing of topical antimicrobials, systemic steroids, anti-parasitic and anti-fungal agents at this time was also recorded.

To allow estimation of pyoderma prevalence the VetCompass database was queried to determine the total number of unique canine patients presented to participating practices for any reason during 2010. This was used as denominator data in calculations estimating the prevalence of diagnosis with at least one pyoderma episode in UK dogs presented to first opinion practice during the year 2010. Prevalence and the 95% CIs were calculated by standard methods [41].

### Additional clinical data from a random subset of study dogs

Random number generation (using Microsoft Excel (12.0) [Microsoft Corporation, Redmond, WA, USA]) was used to select a subset sample of at least 10% of the dogs identified as pyoderma cases by the initial search criteria. These selected cases were reviewed in depth, including evaluation of disease and treatment details, follow-up of clinical progression and validation of the initial case-finding approach. Queries were designed to extract all clinical entries associated with these dogs from the original database on dates up to and including the 31st December 2011. Chronological review of all available free-text clinical history was undertaken for each dog to assess the validity of selection as a pyoderma case and to describe pyoderma lesion depth, underlying cause and treatment-related information in greater detail.

Pyoderma depth was categorised as either ‘surface’ , ‘superficial’ , ‘deep’ , or ‘mixed’ based on lesion description statements recorded by the examining clinician [8]. Cases were classified into one of three groups with respect to diagnosis of a primary cause: if at least one primary disease diagnosis was explicitly stated in clinical notes, based on reported diagnostic tests, prior diagnostic history or (in the absence of these data), strongly expressed suspicion of the managing clinician after clinical examination) classification was ‘confirmed diagnosis of primary condition/s’, and specific conditions were recorded. Cases with no stated primary disease diagnosis and no evidence for performance of relevant diagnostic testing were classed as having either ‘suggested primary cause/s only’ or having ‘no stated or suggested primary cause’.

Additional treatment-related data extracted for this subset included the daily dosing instructions and total number of units (tablets/capsules/volume of injectable formulation) of any initially prescribed systemic antimicrobial agent dispensed across all consecutive prescribing events during the studied pyoderma episode. Based on calculations using patient weight (as recorded on the date of diagnosis) and drug manufacturers’ recommended daily dose (MRDD) rates for treatment of canine pyoderma [42], dogs were classified as having been prescribed at least the minimum MRDD or a dose below this level. Where total duration of prescribed treatment with the systemic antimicrobial agent of interest was not explicitly stated, a theoretical total course length based on units dispensed and dosing instructions was calculated whenever possible.

Non-antimicrobial treatments prescribed or administered to study dogs concurrently with the systemic antimicrobial agent of interest were also recorded. Where appropriate, prescribed products were classified (based on active ingredients, formulation, instructions for use when prescribed and clinical opinion of a board certified veterinary dermatologist) into one or more of the following categories, highlighted due to their potential relevance in cases of skin infection: Systemic steroids, antifungal agents and anti-ectoparasitic agents^d^. Concurrent prescription of shampoos or liquid washes (including non-medicated products) was noted to reflect the intention for regular physical bathing of the skin and coat.

### Statistical analysis

Data were cleaned in Microsoft Excel (version 12.0; Microsoft Corporation, Redmond, WA, USA). Statistical analysis was carried out using Stata (version 11.0; Statacorp, Texas, USA). Variables were categorised where required. Descriptive statistics were calculated for all study dogs with respect to signalment factors and antimicrobial product/s prescribed within 24 hours of recorded diagnosis of the pyoderma episode of interest.

Additional descriptive statistics were calculated for the randomly selected subset of pyoderma cases described in greater detail, including lesion depth, duration of systemic antimicrobial treatment prescribed and adequacy of prescribed daily dose compared with MRDD. The extent of missing or insufficiently specific data was summarized. Mean and standard deviation or median and range were used for the description of quantitative data as appropriate while qualitative data were described using frequencies with percentage of stated totals.

## Endnotes

^a^RVC Ethics Committee, March 2009.

^b^The recording format of the PMS used by participating practices included opportunities for standardised terminology based on the VeNom codes.

^c^Study protocol included a list of terminology accepted as compatible with pyoderma for study purposes, based on clinical codes defined by the VeNom Coding group. Some notable exceptions were also specified, e.g. pyotraumatic dermatitis.

^d^Full category listings are provided in Additional file 1.

## Additional file

Additional file 1:
**Classification of therapeutic products prescribed concurrently with systemic antimicrobial treatment.** Description of data: Classification of prescribed products into groups of particular potential relevance in cases of canine skin infection (based on active ingredients, formulation, instructions for use when prescribed and clinical opinion of a specialist, board certified veterinary dermatologist).

## Abbreviations

EPR: Electronic patient record
PMS: Practice management system
RVC: Royal Veterinary College
RCVS: Royal College of Veterinary Surgeons
FTP: File transfer protocol
SQL: Structured query language
VeNom: Veterinary nomenclature
ID: Identification
CIs: Confidence intervals
MRDD: Manufacturers’ recommended daily dose
TMPS: Trimethoprim sulphonamide
