# Supplementary material for: Prescribing practices of primary-care veterinary practitioners in dogs diagnosed with bacterial pyoderma
Source: BMC Vet Res. 2014 Oct 8;10:240. doi: 10.1186/s12917-014-0240-5 (PMC4193143; doi:10.1186/s12917-014-0240-5)
Supplement: Additional file 1: — Classification of therapeutic products prescribed concurrently with systemic antimicrobial treatment. Description of data: Classification of prescribed products into groups of particular potential relevance in cases of canine skin infection (based on active ingredients, formulation, instructions for use when prescribed and clinical opinion of a specialist, board certified veterinary dermatologist). [file 12917_2014_240_MOESM1_ESM.pdf]

Additional file 1

Classification of therapeutic products prescribed concurrently with systemic antimicrobial treatment

Classification of prescribed products into groups of particular potential relevance in cases of canine skin infection (based on active ingredients, formulation, instructions for use when prescribed and clinical opinion of a specialist, board certified veterinary dermatologist).

**Products classified as anti-ectoparasite agents**	**Commercial product details (Manufacturer, location)**
Advocate spot-on	Bayer Animal Health, Leverkusen (Germany)
Aludex topical solution	Merck Animal Health, Milton Keynes (UK)
Frontline Combo spot-on	Merial Animal Health, Harlow (UK)
Frontline spot-on	Merial Animal Health, Harlow (UK)
Interceptor (oral)	Novartis Animal Health, Camberley (UK)
Panomec (oral)	Merial Animal Health, Harlow (UK)
Stronghold spot-on	Zoetis, London (UK)
Unspecified flea adulticide	*Unknown*
Unspecified oral ivermectin	*Unknown*
Unspecified oral milbemycin	*Unknown*

**Products classified as topical antimicrobial agents**	**Commercial product details (Manufacturer, location)**
Aurizon topical drops	Vetoquinol UK Ltd, Buckingham (UK)
Chloramphenicol topical drops	*Unknown*
Etiderm shampoo	Virbac Animal Health, Bury St Edmunds (UK)
Flamazine ointment	King Pharmaceuticals (USA)
Fuciderm ointment	Dechra Veterinary Products, Shrewsbury (UK)
Fucidin ointment	Leo Pharma Inc, Otario (USA)
Hibiscrub solution	*Unknown*
Malacetic wipes, shampoo or spray	Dechra Veterinary Products, Shrewsbury (UK)
Malaseb shampoo	Dechra Veterinary Products, Shrewsbury (UK)
Otomax topical drops	Schering-Plough, New Jersey (USA)
Paxcutol shampoo	Virbac Animal Health, Bury St Edmunds (UK)
Tiacil ointment	Virbac Animal Health, Bury St Edmunds (UK)

Additional file 1 (continued)

**Products classified as systemic steroidal agents**	**Commercial product details (Manufacturer, location)**
Medrone oral tablets	Pfizer Ltd, Sandwich (UK)
Unspecified oral dexamethasone	*Unknown*
Unspecified oral prednisolone	*Unknown*

**Products classified as anti-fungal agents**	**Commercial product details (Manufacturer, location)**
Aurizon topical drops	Vetoquinol UK Ltd, Buckingham (UK)
Itrafungol oral solution	Elanco, Basingstoke (UK)
Malacetic wipes, shampoo or spray	Dechra Veterinary Products, Shrewsbury (UK)
Malaseb shampoo	Dechra Veterinary Products, Shrewsbury (UK)
Nizoral oral tablets	Janssen Pharmaceuticals, Turnhoutseweg (Belgium)
Sporanox oral capsules	Janssen Pharmaceuticals, Turnhoutseweg (Belgium)
Unspecified oral itraconazole	*Unknown*
